# Elevated expression of the adhesion GPCR ADGRL4/ELTD1 promotes endothelial sprouting angiogenesis without activating canonical GPCR signalling

**DOI:** 10.1038/s41598-021-85408-x

**Published:** 2021-04-23

**Authors:** David M. Favara, Ines Liebscher, Ali Jazayeri, Madhulika Nambiar, Helen Sheldon, Alison H. Banham, Adrian L. Harris

**Affiliations:** 1 Balliol College, University of Oxford, Oxford, OX1 3BJ UK; 2 Department of Oncology and Weatherall Institute of Molecular Medicine, University of Oxford, Oxford, OX3 7DQ UK; 3 Rudolf Schönheimer Institute of Biochemistry, Department of Molecular Biochemistry, University of Leipzig, 04103 Leipzig, Germany; 4 Heptares Therapeutics Ltd, Welwyn Garden City, AL7 3AX UK; 5 Nuffield Division of Clinical Laboratory Science, Radcliffe Department of Medicine, University of Oxford, Oxford, OX3 9DU UK; 6 Present Address: Cambridge University Hospitals NHS Foundation Trust and Department of Oncology, Cambridge University, Cambridge, CB2 0XZ UK; 7 Present Address: OMass Therapeutics, Oxford, OX4 4GE UK; 8 Present Address: Sosei Heptares, Cambridge, CB21 6DG UK

**Keywords:** Cell biology, Cell signalling

## Abstract

ADGRL4/ELTD1 is an orphan adhesion GPCR (aGPCR) expressed in endothelial cells that regulates tumour angiogenesis. The majority of aGPCRs are orphan receptors. The *Stachel* Hypothesis proposes a mechanism for aGPCR activation, in which aGPCRs contain a tethered agonist (termed *Stachel*) C-terminal to the GPCR-proteolytic site (GPS) cleavage point which, when exposed, initiates canonical GPCR signalling. This has been shown in a growing number of aGPCRs. We tested this hypothesis on ADGRL4/ELTD1 by designing full length (FL) and C-terminal fragment (CTF) ADGRL4/ELTD1 constructs, and a range of potential *Stachel* peptides. Constructs were transfected into HEK293T cells and HTRF FRET, luciferase-reporter and Alphascreen GPCR signalling assays were performed. A stable ADGRL4/ELTD1 overexpressing HUVEC line was additionally generated and angiogenesis assays, signalling assays and transcriptional profiling were performed. ADGRL4/ELTD1 has the lowest GC content in the aGPCR family and codon optimisation significantly increased its expression. FL and CTF ADGRL4/ELTD1 constructs, as well as *Stachel* peptides, did not activate canonical GPCR signalling. Furthermore, stable overexpression of ADGRL4/ELTD1 in HUVECs induced sprouting angiogenesis, lowered in vitro anastomoses, and decreased proliferation, without activating canonical GPCR signalling or MAPK/ERK, PI3K/AKT, JNK, JAK/HIF-1α, beta catenin or STAT3 pathways. Overexpression upregulated *ANTXR1*, *SLC39A6*, *HBB*, *CHRNA*, *ELMOD1*, *JAG1* and downregulated *DLL4*, *KIT*, *CCL15*, *CYP26B1*. ADGRL4/ELTD1 specifically regulates the endothelial tip-cell phenotype through yet undefined signalling pathways.

## Introduction

ADGRL4/ELTD1 is an orphan adhesion GPCR which is expressed in endothelial cells^1,2^ and smooth muscle cells^3^ where it regulates both physiological and tumour angiogenesis^4^. Its expression is induced by VEGFR and TGF-β^4,5^ and repressed by DLL4^4^. Disruption of ADGLR4/ELTD1 expression reduced endothelial sprouting in vitro and caused vascular abnormalities incompatible with life in zebrafish embryos^4^. In mice, embryonic ADGRL4/ELTD1 knockout does not cause significant vascular defects^6,7^ which is thought to be due to the presence of additional genetic redundancy through an enlarged angiogenesis-gene repertoire in higher vertebrates^8^. ADGRL4/ELTD1 is overexpressed, compared to normal adjacent endothelium, in tumour-associated endothelial cells in several common tumour types (head and neck, renal, colorectal, ovarian and brain cancers)^4,5^ as well as in glioblastoma tumour cells^9^. It is not expressed in the majority of tumour cell lines^10^. Systemic vascular ADGRL4/ELTD1 silencing in mice implanted with human tumour xenografts (colorectal and ovarian cancers) decreased microvascular density and induced tumour shrinkage with increased survival without a detrimental systemic vascular effect^4^. In glioblastoma tumour cells, ADGRL4/ELTD1 silencing led to improved survival^9,11,12^. Paradoxically, high ADGRL4/ELTD1 expression in patients treated with systemic anti-cancer treatment correlated with improved overall survival (head and neck squamous carcinoma, renal, colorectal and ovarian cancers)^4^. A potential explanation is that ADGRL4/ELTD1 is associated with a vascular stabilising effect with better tumoural drug delivery. This makes ADGRL4/ELTD1 a complex target to consider for therapy.

Like the majority of adhesion GPCRs, ADGRL4/ELTD1’s activation and signalling mechanisms remain unknown^13^. The *Stachel* hypothesis proposes that the majority of aGPCR_S_ contain a tethered agonist (the *Stachel*) C-terminal to the GPCR-proteolysis site (GPS) cleavage point which, when exposed following N-terminal fragment (NTF) modification (such as ligand binding to the NTF) is able to strike the receptor’s extracellular seven transmembrane (7TM) loops and initiate canonical GPCR signalling^14^. Since its publication, the *Stachel* hypothesis has been employed to solve the canonical GPCR signalling and activation of a growing number of aGPCRs: ADGRG6/GPR126, ADGRD1/GPR133^14^, ADGRG1/GPR56, ADGRF1/GPR110^15^, ADGRG5/GPR114^16^, ADGRG2/GPR64^17^, ADGRL1/LPHN1^18^, ADGRB1/BAI1, ADGRF5/GPR116^19^, ADGRL2/LPHN2^20^. We tested whether the *Stachel* hypothesis applied to ADGRL4/ELTD1, and investigated the effect of ADGRL4/ELTD1 upregulation on angiogenesis.

## Results

### Codon optimisation increased ADGRL4/ELTD1 expression

**Figure 1:**
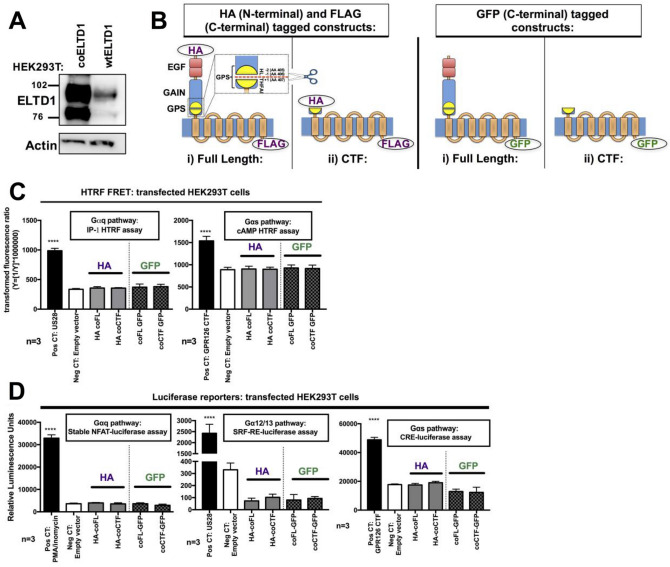
Codon optimisation increases ADGRL4/ELTD1 expression but FL and CTF forms of ADGRL4/ELTD1 do not activate canonical GPCR signalling. (**A**) Representative Western blot of co FL ADGRL4/ELTD1 compared to wt FL ADGRL4/ELTD1. (**B**) ADGRL4/ELTD1 signalling construct design showing FL and CTF forms with different tagging strategies. (**C**) Gαq HTRF FRET assays in ADGRL4/ELTD1 transiently transfected HEK293T cells. Positive controls used were US28 for Gαq (*p* < 0.0001) and the CTF form of ADGRLG6/GPR126 for Gαs (*p* < 0.0001) (**D**) Gαq, Gα12/13, Gαs luciferase assays in ADGRL4/ELTD1 transiently transfected HEK293T cells. Positive controls used were PMA/ionomycin for Gαq (*p* < 0.0001), US28 for Gα12/13 (*p* < 0.0001) and the CTF form of GPR126 for Gαs (*p* < 0.0001). (Abbreviations: Pos CT = positive control; Neg CT = negative control; FL = full length; wt = wild type; co = codon optimised; CTF = C-terminal fragment; HA = hemagglutinin; GFP = green fluorescent protein).

After experiencing difficulties overexpressing the ADGRL4/ELTD1 protein (Fig. 1A) we observed that ADGRL4/ELTD1’s nucleotide sequence has a GC content of 34% (codon adaptation index 0.18), the lowest within the adhesion and secretin GPCR families (Figure S1A). The average GC content for coding sequences in *Homo sapiens* is 52%^21^. Wild type (WT) full length (FL) *ADGRL4/ELTD1* was codon optimised (co) retaining its amino acid coding sequence and increasing its GC content to 58% (codon adaption index 0.76), a level higher than the majority of compared receptors (Figure S1A) with the resulting co FL ADGRL4/ELTD1 producing a much higher level of protein expression compared to the original sequence when transfected into HEK293T cells. (Fig. 1A). Codon optimised versions of ADGRL4/ELTD1 were then used for all subsequent experiments.

### ADGRL4/ELTD1 construct design and expression

Two recombinant forms of co ADGRL4/ELTD1 were created to test the tethered agonist hypothesis^14^: (1) a FL form—showing more or less basal activity; and (2) a C-terminal fragment (CTF) mutant, where everything N-terminal to the GPS consensus cleavage site (amino acids HL//T^22,23^) was removed—which was shown to induce constitutive activity in aGPCRs (Fig. 1B). To minimise the risk of tags interfering with signalling, two series of constructs were designed with different tagging strategies: one contained both N-terminal HA and C-terminal FLAG tags, as used in the original studies^14,24,25^, whilst a second contained only a C-terminal GFP tag (Fig. 1B). Wild type ADGRL4/ELTD1’s signal peptide was included in all recombinant versions created (both FL and CTF forms).

Each was transiently transfected into HEK293T cells and assessed for expression of the appropriate protein tag at 48 h using FACS. The N-terminal HA tagged FL and CTF constructs were expressed on the cell surface with the FL construct having a higher surface expression than the CTF construct (55% vs. 30% positivity) (Figure S1B). FACS results for the whole cell C-terminally GFP tagged FL and CTF constructs showed equal overall fluorescence for both constructs (Figure S1B) that was greater than the WT equivalent (Figure S1C). Similar results for cell surface expression were obtained using an ‘in-house’ anti-ADGRL4/ELTD1 monoclonal antibody raised against the NTF^4^ (Figure S1C).

Confocal microscopy also showed surface and cytoplasmic GFP expression for both constructs (Figure S1D). Surface expression of ADGRL4/ELTD1’s N-terminal HA tagged constructs was comparable to that of HA tagged FL and CTF ADGRG6/GPR126^14^ as well as HA tagged P2Y12 (a Rhodopsin-like GPCR with very high surface expression) (Figure S1E).

### Neither full length nor CTF ADGRL4/ELTD1 or ADGRL4/ELTD1’s *Stachel* peptides activate canonical GPCR signalling

GPCR signalling assays were performed using three experimental approaches (see methods), assessing second messenger products or transcription factors downstream of specific G-protein pathway coupling. As all of these assays are accumulation assays, they were performed at 48 h following transfection to allow sufficient time for high levels of ADGRL4/ELTD1 surface expression.

HTRF FRET assays in transiently transfected HEK293T cells showed no Gαq or Gαs signalling activity from either FL or CTF ADGRL4/ELTD1 compared to an empty vector control (Fig. 1C), despite adequate cell surface expression and positive controls. Luciferase reporter assays (downstream of a wider range of G proteins) in similarly transfected HEK29T cells showed no sign of canonical GPCR signalling activity, despite proper positive controls (Fig. 1D), as did the Alphascreen system (Figure S1F). In summary neither FL nor CTF ADGRL4/ELTD1 constructs (in differently tagged configurations) elicited any canonical GPCR signalling, despite adequate and appropriate localisation of expression, when compared to controls under conditions where signalling was observed for other aGPCRs.

**Figure 2:**
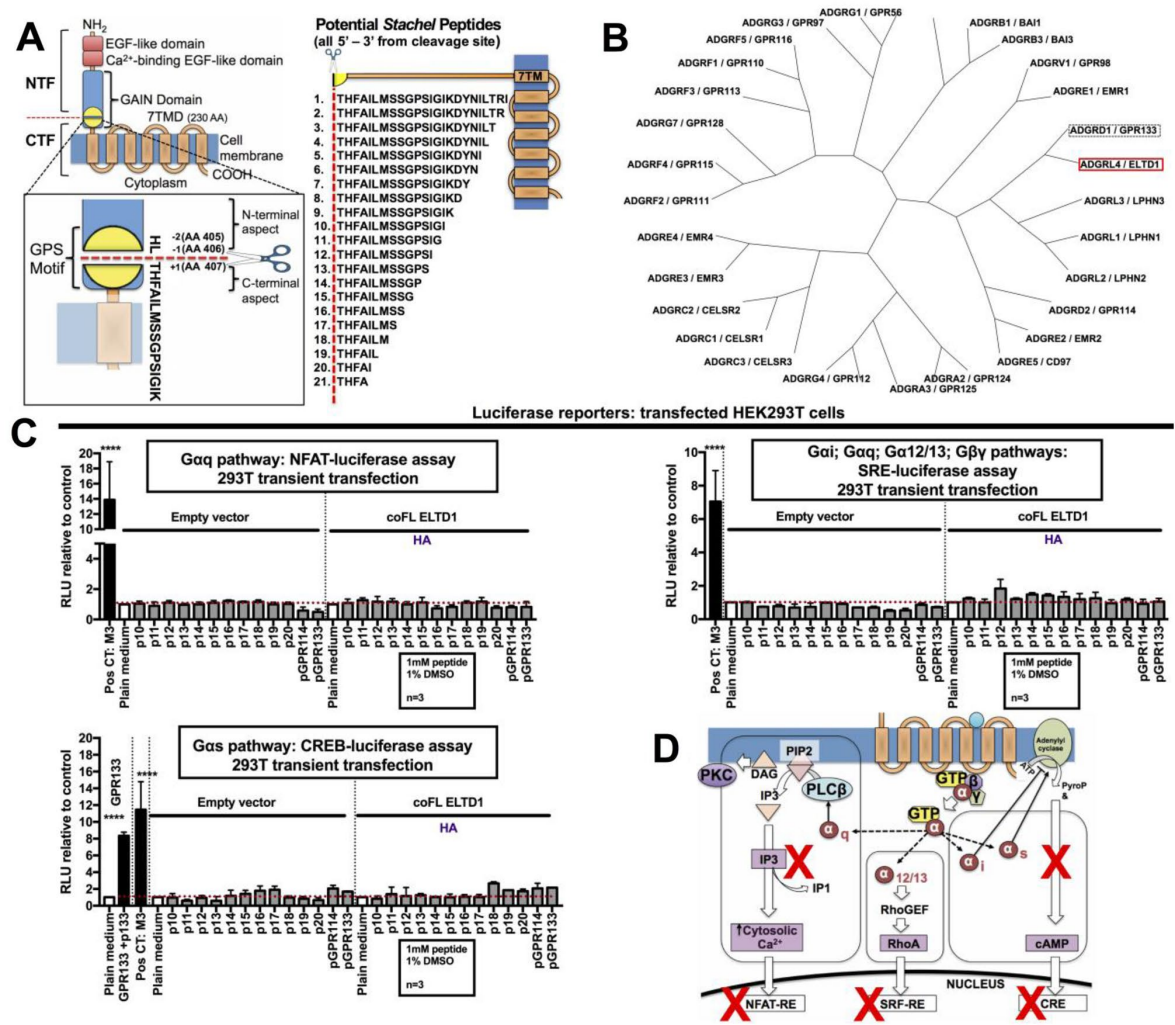
ADGRL4/ELTD1’s putative *Stachel* peptides do not activate canonical GPCR signalling. (**A**) Design of the 21 putative ADGRL4/ELTD1 *Stachel* peptides. (**B**) Radial cladogram comparing similarity of all aGPCR GPS domains. (**C**) Gαq, Gαs, Gαi, Gα12/13, Gβγ *Stachel* peptide luciferase assays in HEK293T cells transfected with co FL ADGRL4/ELTD1. Positive controls used were the CHRM3/M3 GPCR receptor exposed to 10 µM carbachol for Gαq, Gαs, Gαi, Gα12/13, Gβγ (*p* < 0.0001) and GPR133 exposed to its *Stachel* peptide (p133) for Gαs (*p* < 0.0001). (**D**) Graphical summary of canonical GPCR signalling pathways investigated in this study. (Abbreviations: Pos CT = positive control; co = codon optimised; FL = full length; NTF = N-terminal fragment; CTF = C-terminal fragment).

We also investigated whether a peptide analogous to ADGRL4/ELTD1’s putative tethered agonist region could elicit canonical GPCR signalling. 21 peptides of varying length corresponding to the amino acid sequence beginning at ADGRL4/ELTD1’s consensus GPS cleavage site and extending C-terminally to the predicted start of the first 7TM loop (Fig. 2A) were tested. Peptides were generally highly insoluble. After preliminary optimisation and screening studies with all 21 peptides, subsequent studies focussed on peptides p10-20 (Fig. 2A) as most published *Stachel* peptides had been of a length within this range. The 11 selected ADGRL4/ELTD1 *Stachel* peptides were then assayed for activation of co FL ADGRL4/ELTD1 transiently transfected in HEK293T cells using luciferase assays corresponding to all canonical GPCR signalling pathways. With peptide insolubility being of concern we wanted to include some additional peptide controls. Thus, phylogeny comparisons were conducted on the predicted GPS sequences of all human aGPCR family members to find the relative with the highest GPS similarity to ADGRL4/ELTD1’s GPS (ADGRA1/GPR123 does not contain a GPS domain and was not included) as it has been shown that aGPCRs that cannot be activated through their own tethered agonist can be induced through closely related *Stachel* peptides^26^. ADGRD1/GPR133 had the most similar GPS (Fig. 2B) and ADGRG5/GPR114’s peptide (pGPR114) (Wilde et al., 2016) was chosen as an additional control peptide. Neither of these peptides showed Gαq, Gαs, Gαi, Gα12/13, or Gβγ pathway signalling activity with ADGRL4/ELTD1 (Fig. 2C).

Results from HTFR FRET assays performed in similarly transfected HEK293T cells as well as on non-transfected HUVECS (which constitutively express WT FL ADGRL4/ELTD1) using all 21 *Stachel* peptides showed no Gαs or Gαi signalling (Figure S2A-B). In summary, ADGRL4/ELTD1 did not appear to activate any form of canonical GPCR signalling (Fig. 2D).

### ADGRL4/ELTD1 overexpression induces sprouting angiogenesis and regulates the endothelial tip-cell phenotype

**Figure 3:**
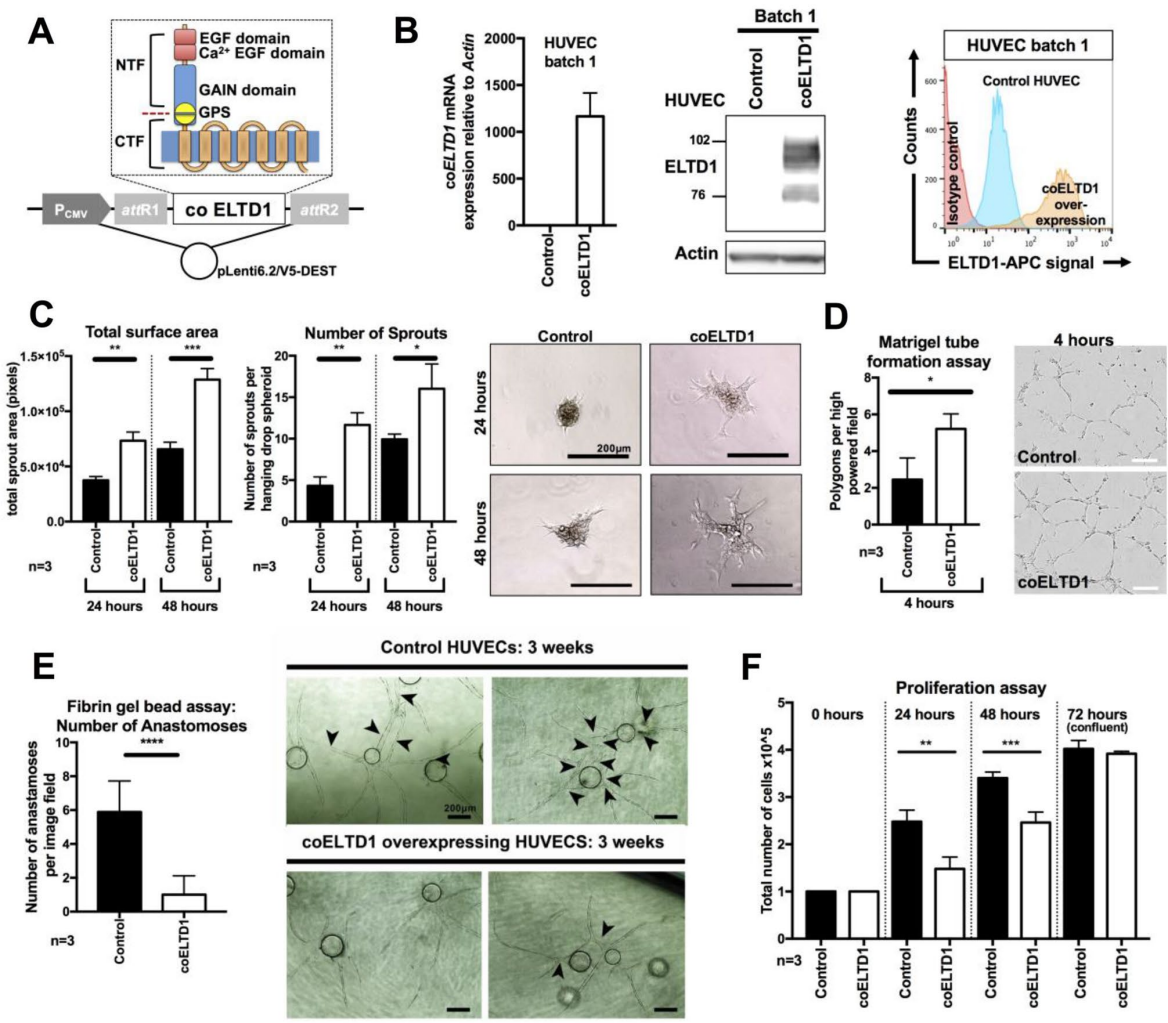
Co FL ELTD1/ADGRL4 stable overexpression in HUVECS induces the tip cell phenotype. (**A**) Illustration of full length codon optimised ADGRL4/ELTD1 within the pLenti6.2/V5-DEST lentiviral vector. (**B**) Stable overexpression of co FL ADGRL4/ELTD1 in HUVECs (batch 1) showed increased transcripts (qPCR), total protein (Western blot) and cell surface expression (FACS) compared to control HUVECs. HUVECS overexpressing co FL ADGRL4/ELTD1 showed (**C**) increased total spheroid surface area and number of sprouts, compared to controls at both 24 and 48 h; (**D**) increased capillary tube formation; (**E**) decreased number of vascular anastomoses/branches between adjacent vessels (arrowheads point to anastomotic branches); (**F**) reduced HUVEC proliferation.

Although ADGRL4/ELTD1 knockdown has previously been shown to inhibit sprouting angiogenesis in HUVECs^4^, the effect of overexpression remains unknown. This is biologically relevant to disease pathology as ADGRL4/ELTD1 is upregulated in endothelial cells found in the tumour microenvironment^4^. Functionality of the overexpressed co ADGRL4/ELTD1 protein was investigated in different batches of stably transduced low passage primary endothelial cells (Fig. 3A,B, Figure S3A,B) which constitutively express WT ADGRL4/ELTD1 at a low level. Wild type *ADGLR4/ELTD1* mRNA expression (distinguishable from co *ADGLR4/ELTD1* at the transcript level) was similar for both control and co ADGRL4/ELTD1 transduced samples, showing that ADGRL4/ELTD1 protein overexpression did not exert negative feedback onto wild type *ADGLR4/ELTD1* expression (Figure S3C).

ADGRL4/ELTD1 overexpression in HUVECs induced sprouting angiogenesis (increased total spheroid surface area [24 h *p* = 0.002; 48 h *p* = 0.0008], increased the number of sprouts [24 h *p* = 0.002; 48 h *p* = 0.03] (Fig. 3C) and increased endothelial capillary tube formation (*p* = 0.03) (Fig. 3D). The fibrin gel bead assay showed that ADGRL4/ELTD1 overexpression inhibited vessel branching reducing vascular anastomoses/branches between adjacent vessels at the 3-week conclusion of the assay (*p* < 0.00001) (Fig. 3E). In both groups, lumen formation appeared to be similar (Fig. 3E). HUVEC proliferation was decreased with 40% fewer cells at 24 h (*p* = 0.01) and 28% fewer cells at 48 h (*p* = 0.01) (Fig. 3F). There was no effect on endothelial cell migration (start of assay *p* NS; end of assay at 12 h *p* NS) (Figure S3D), or adhesion to various angiogenesis-associated extracellular matrix components (collagen I, collagen IV, laminin, vitronectin, fibronectin *p* NS for all) (Figure S3E) and matrigel (*p* NS) (Figure S3F).

**Figure 4:**
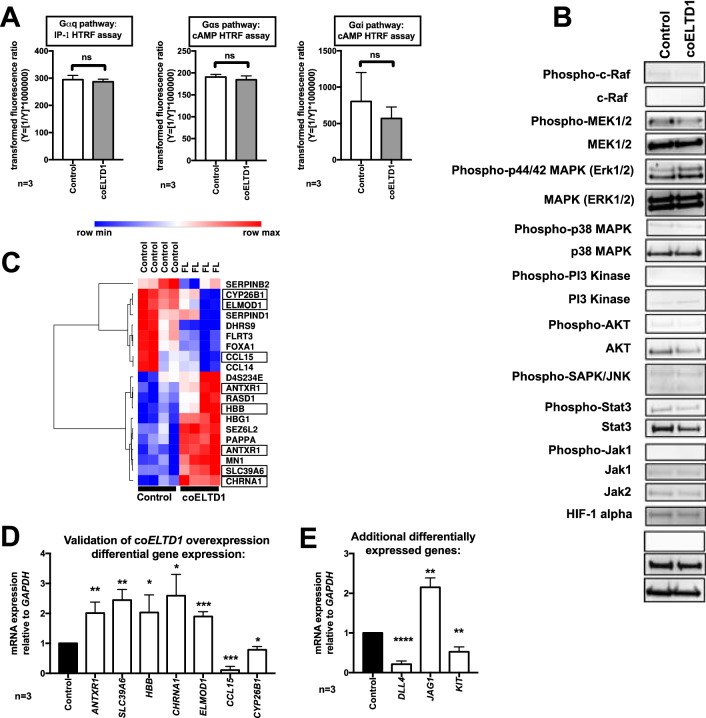
ADGRL4/ELTD1 overexpression in HUVECS does not induce canonical GPCR or MAPK/ERK, PI3K/AKT JNK, JAK/HIF-1α, Beta-catenin and STAT3 signalling. (**A**) Gαq, Gαs, Gαi HTRF FRET assays in ADGRL4/ELTD1 overexpressing HUVECs showed no signalling. (**B**) Western blot showed no change in a range of non-GPCR signalling pathways: MAPK/ERK, PI3K/AKT JNK, JAK/HIF-1α, Beta-catenin and STAT3 pathways. (**C**) Heatmap depicting significant differentially expressed genes following transcriptional profiling in FL ADGRL4/ELTD1 stable overexpressing HUVEC cells compared to control HUVECS. The intensity colouring (low = blue; high = red) represents the z-score for each gene. Boxes represent validated genes. (**D**) qPCR validation showed *ANTXR1*, *SLC39A6*, *HBB*, *CHRNA*, *ELMOD1* upregulation, and *CCL15* and CYP26B1 downregulation. (**E**) qPCR of additional genes showed *DLL4* and *KIT* downregulation and *JAG1* upregulation. (Abbreviations: co = codon optimised; FL = full length).

Gαq, Gαs, Gαi HTRF FRET assays were performed in both ADGRL4/ELTD1 overexpression and control HUVECs and showed no significant induction of any Gαq, Gαs, or Gαi signalling (*p* NS for all) (Fig. 4A). Western blotting revealed no activation of the MAPK/ERK, PI3K/AKT JNK, JAK/HIF-1α, beta catenin, NOTCH and STAT3 pathways in ADGRL4/ELTD1 overexpressing HUVECs (Fig. 4B).

Microarray transcriptional profiling was performed on 2 batches of ADGRL4/ELTD1 overexpression and control HUVECS performed in duplicate (Fig. 4C) to investigate potential changes in gene expression that might be responsible for the functional changes in endothelial cell biology. This showed significant upregulation of *ANTXR1* (*p* = 0.009), *SLC39A6* (*p* = 0.002), *HBB* (*p* = 0.04), *CHRNA* (*p* = 0.02) *ELMOD1* (*p* = 0.0006), and downregulation of *CCL15* (*p* = 0.0003) and *CYP26B1* (*p* = 0.03) (Fig. 4D). Overexpressed FL codon optimised ADGRL4/ELTD1 was not detected as the microarray only had probes for wild type ADGRL4/ELTD1. A further panel of genes recently shown to be upregulated by ADGRL4/ELTD1 knockdown in HUVECS^27^ were also tested, showing downregulation of *DLL4* (*p* < 0.0001), and *KIT* (*p* = 0.003) and upregulation of *JAG1* (*p* = 0.001) (Fig. 4E).

## Discussion

We investigated ADGRL4/ELTD1 receptor signalling and found that ADGRL4/ELTD1 had the lowest GC content in the aGPCR family, did not show canonical GPCR signalling, and induced an endothelial tip-cell phenotype when overexpressed (as occurs in tumour-associated endothelium^28^). The finding that wildtype ADGRL4/ELTD1’s GC content was the lowest in the aGPCR family is interesting from a functional and evolutionary perspective. Codon usage regulates mRNA stability and translational efficiency with optimal codons associated with abundant tRNAs for the cognate codon, and higher levels of protein expression, whilst those with low GC content and non-optimal codons are associated with low level expression^29–31^. ADGLR4/ELTD1’s non-optimal codon usage suggests that it has been evolutionary beneficial for endothelial and smooth muscle cells to tightly control ADGRL4/ELTD1 expression, despite natural selection generally opting for more optimal codon usage across the genome^32^. An example of this are the genes of the cell cycle, which have a low GC and non-optimal codon usage as a mechanism to tightly control expression^33^.

A recent publication suggested that ADGRL4/ELTD1 signals via the JAK/STAT3/HIF-1α pathway in glioblastoma to regulate proliferation and migration^34^ although this is not a known direct cascade for GPCRs. We did not observe any downstream signalling events in HEK293T, COS-7 or HUVEC cells, indicating that the interaction between ADGRL4/ELTD1 and the JAK/STAT3/HIF-1α pathway is specific for glioblastoma or other cancer cell types and does not apply within endothelial cells. Currently, the direct intracellular consequences of ADGRL4/ELTD1 activation remain unknown. This lack of signalling could potentially be due to: (1) lack of the endogenous *Stachel* sequence or insufficient stimulus for its exposure, (2) G protein-independent signalling (3) function via cell surface interactions.

ADGRB1/BAI1 and ADGRG1/GPR56 are two aGPCR_S_ which induce G protein-coupled signalling through both the tethered agonist as well as through a non-tethered agonist mechanism^35^. For both receptors, both their CTF forms as well as a truncated form of the 7TM (without a tethered agonist sequence) signal constitutively but also differentially. For ADGRG1/GPR56, its CTF induced greater Gα_12/13_ and Gα_q_ signalling than its 7TM form, whilst for ADGRB1/BAI1, its CTF induced greater Gα_12/13_ signalling whilst its 7TM form induced greater Gα_q_ signalling^35^. However, even when *Stachel*-independent signalling was detected it did not reveal a signal that was absent in full length or *Stachel*-containing constructs. Thus, we believe it rather unlikely that 7TM constructs would reveal any signal in the investigated assays.

A few aGPCRs such as ADGRB1/BAI1^36^ and ADGRB3/BAI3^37^ mediate G protein-independent signalling via PDZ binding motifs on their intracellular portions^36–38^. ADGRB1/BAI1 can also activate RAC1 signalling through interactions with TIAM1, another regulator of RAC1^39^.

Although ADGRL4/ELTD1 lacks a PDZ motif, interestingly its overexpression induced the expression of another ELMO protein family member, *ELMOD1*. ELMOD1 is a GTPase-activating protein, activates the GTP-binding protein ARF6 to regulate actin assembly and membrane trafficking^40^. In endothelial cells, ARF6 is known to regulate angiogenesis, and does so by modulating the trafficking and recycling of VEGFR2 from the cell surface, ensuring adequate VEGFR2 expression^41,42^. Other known G protein-independent aGPCR signalling components include GABPγ associating with ADGRB2/BAI2 and leading to VEGF induction^43^, ADGRC1/Celsr1 forming a complex with Dishevelled, DAAM1 and PDZ-RhoGEF and ADGRA3/GPR125 influencing dishevelled and Wnt signalling^44^.

Another possible explanation for the lack of detectable signalling pathways is that other means of activation need to be employed to activate the receptor. Among them mechanical forces or incubation of the full length receptor with a ligand that has not yet been identified. Further, small molecule compounds have been shown to activate aGPCRs. Examples include beclomethasone dipropionate on GPR97^45^ or Gedunin- and Khivorin-derivatives on GPR56 and GPR114^46^. It is possible that endogenous small molecules could activate ADGRL4/ELTD1 irrespective of the existence of a functional tethered agonist. Also, ADGRL4/ELTD1’s function may act in trans as a ligand for other molecules as has been suggested for ADGRL1/LPHN1^47^ or ADGRB1/BAI1^48^. Future experiments are planned to determine whether ELTD1/ADGRL4’s NTF or 7TM regions are responsible for its angiogenesis phenotype seen when FL ELTD1/ADGRL4 is overexpressed in endothelial cells. This will be performed using two approaches: CTF form overexpression and 7TM-mutant form overexpression. Should CTF overexpression not induce an angiogenesis phenotype, this suggests that ADGRL4/ELTD1’s NTF is central to promoting its angiogenic effect with activity possibly influenced by NTF adhesion. Conversely, if 7TM-mutant overexpression (FL ELTD1 truncated prematurely after the first or second 7TM loop) does not induce an angiogenesis phenotype, this would suggest that ADGRL4/ELTD1’s 7TM is central to promoting its angiogenic phenotype.

Limitations of our signalling analysis include the use of transient transfection and HEK293T cells which may not contain the cellular machinery necessary for ADGRL4/ELTD1 signalling. Despite this, the majority of published aGPCR *Stachel* papers have used these cells for GPCR signalling assays although they do not constitutively express the receptor. ADGRL4/ELTD1’s *Stachel* peptides were all very water insoluble and difficult to dissolve. Various modifications were added to the N- and C- peptide termini to try and improve solubility with no effect. In contrast, some aGPCRs have more water-soluble *Stachel* peptides, for example GPR116’s p116^19,26^. However, *Stachel* peptides of GPR114 or GPR133 are similarly insoluble, yet they yield significant activation on full length and mutant aGPCRs^14,16^.

Our finding that ADGRL4/ELTD1 induced a tip phenotype in endothelial cells is opposite and complementary to the effect seen on ADGRL4/ELTD1 silencing^4^, as is the effect on the core angiogenic Notch genes *DLL4* and *JAG1*, and *KIT*. The finding that ADGRL4/ELTD1 overexpression decreases anastomoses suggests that there may be a failure to switch off the tip cell phenotype as vascular anastomoses occur when two sprouts led by endothelial tip cells meet and merge, with the resultant loss of the tip cell phenotype and induction of the stalk phenotype.

ADGRL4/ELTD1 overexpression also altered patterns of gene expression in HUVECs by inducing *ANTXR1,* SLC39A6, *HBB* and *CHRNA1* and repressing *CCL15*. It is possible that some of the products of these genes may contribute to ADGRL4/ELTD1’s ability to promote angiogenesis. *ANTXR1* is a transmembrane receptor that similarly to ADGRL4/ELTD1 is upregulated in tumour endothelial cells^49^ and which promotes tumour angiogenesis and endothelial proliferation^50^. Similarly, to ADGRL4/ELTD1, *ANTXR1* silencing in xenografts of diverse tumour origin reduced tumour size^51^. SLC39A6 is a zinc transporter associated with the metastasis of pancreatic, oesophageal, prostate and lung cancers^52–54^ however its link to endothelial biology remains unexplored. Interestingly, ADGRL4/ELTD1 silencing was shown to inhibit the metastasis of ovarian cancer xenografts^4^.

*HBB* is the β-subunit of haemoglobin expressed by red blood cells. This is an interesting association considering the link between progenitor endothelial cells which develop into haematopoietic stem cells (HSCs) which produce erythrocytes^55^. The association with the haemogenic endothelium is further strengthened through ADGRL4/ELTD1’s antagonistic relationship with KIT^27^, a positive regulator of haematopoietic stem cells^56^ and the endothelial niche^57^. Furthermore, ADGRL4/ELTD1 (along with ADGRG1/GPR56, another aGPCR) forms part of the transcriptional programme behind the transdifferentiation of haemogenic endothelial cells (HECs) to HSCs, which then produce the cells of the haematopoietic system^58^. This presents an exciting new direction of ADGRL4/ELTD1 research.

ADGRL4/ELTD1 upregulation in the tumour vasculature has been identified as a common finding in several different cancer types, where it has been hypothesised to promote tumour angiogenesis^4^. Here we show that ADGRL4/ELTD1 overexpression in normal primary endothelial cells does indeed have a pro-angiogenic effect. While we could not identify the signalling cascades utilised by ADGRL4/ELTD1, we identified several genes whose expression is altered on ADGRL4/ELTD1 overexpression providing possible mechanistic insights into how it may drive tumour angiogenesis. The further study of such markers related to ADGRL4/ELTD1 function will also provide valuable information for monitoring the efficacy of future therapies aiming to inhibit the expression and/or function of ADGRL4/ELTD1 in the tumour microenvironment.

## Methods

### Codon optimisation

GC content, codon usage analysis and optimisation was performed using JCat^59^ and Biomart^60^. We transiently transfected both codon optimised (co) and wild type (WT) forms of full length (FL) ADGRL4/ELTD1 into HEK293T cells (which do not constitutively express ADGRL4/ELTD1) and analysed total cell ADGRL4/ELTD1 protein expression after 48 h. HEK293T cells were selected as the majority of aGPCR signalling experiments to date have been performed in these cells.

### ADGRL4/ELTD1 construct design

Conserved domains were mapped onto the ADGRL4/ELTD1 amino acid sequence using two predictive algorithms: SMART 2015^61^ and NCBI Conserved Domain Search^62^. SignalP 4.0^63^ was used to determine the signal peptide. The CTF construct only contained ADGRL4/ETLD1’s sequence C-terminal to the GPS cleavage site (amino acids HL//THFAI) in addition to the signal peptide. Constructs were synthesised by Biomatik and then cloned into pcDNA3.1 plasmid vectors and verified by sequencing.

### ADGRL4/ELTD1 putative *Stachel* peptide design

Peptides analogous to the C-terminal end of the GPS cleavage site were designed, extending to the first predicted loop of the 7TM. Peptides were produced to a high purity (> 90%) by Biomatik. Peptides containing additional water-soluble molecules, in an attempt to increase solubilisation (biotin, PEG2, betaine and amino acids KKRR), were synthesised by Cambridge Research Biochemicals. Peptide sequences are listed in Supplementary Methods Table 1.

### Cell lines and maintenance

All cells, including cell biology assays using HUVECs, were cultured in tissue culture incubators at 37 °C with a humidified atmosphere of 5% CO_2_ and 95% filtered atmospheric air; unless otherwise indicated. HUVECS pooled from multiple donors (Lonza) were cultured in EBM-2 (Lonza) supplemented with EGM-2 (Lonza), changed every 48 h. HEK293T (Thermo Scientific) and GloResponse NFAT-RE-luc2P HEK293 (Promega) cells were cultured in DMEM supplemented with 10% FCS and 1% L-glutamine (Lonza), changed every 48 h.

### Western blotting

Proteins in cell lysates were quantified, resolved by SDS-PAGE and then transferred to a PVDF membrane (Millipore). After blocking for 1 h in low fat milk, primary antibodies were added overnight at 4 °C. Primary antibodies: ELTD1 (Sigma HPA025229) 1:1000; β-actin conjugated to HRP (Sigma 3854) 1:10,000; Akt (Cell Signaling 9272) 1:1000; Phospho-Akt (Cell Signaling 4060) 1:1000; Beta Catenin (Cell Signaling 8480) 1:1000; Cleaved Notch1 (Cell Signaling mAb 4147) 1:1000; c-Raf (Cell Signaling 53,745) 1:1000; Phospho-c-Raf (Cell Signaling 9427) 1:1000; HIF-1α (Novus Biologicals NB100-105SS) 1:1000; Phospho-Jak1 (Cell Signaling 66,245) 1:1000; Jak1 (Cell Signaling 3344) 1:1000; Jak2 (Cell Signaling 3230) 1:1000; MEK1/2 (Cell Signaling 4694) 1:1000; Phospho-MEK1/2 (Cell Signaling 9154) 1:1000; PI3 Kinase (Cell Signaling 4257) 1:1000; Phospho-PI3 Kinase (Cell Signaling 4228S) 1:1000;Phospho-SAPK/JNK (Cell Signaling 9251) 1:1000; p38 MAPK (Cell Signaling 8690) 1:1000; Phospho-p38 (Cell Signaling 4511) 1:1000; Phospho-p44/42 MAPK (Erk1/2) (Cell Signaling 4370) 1:1000; Stat3 (Cell Signaling 4904) 1:1000; Phospho-Stat3 (Cell Signaling 9145) 1:1000. After washing, a secondary antibody: Anti-rabbit conjugated to HRP (DAKO P044801-2) 1:2500 was applied. Membrane immunoreactivity was detected using ECL Prime (GE Healthcare), visualisation was performed using an ImageQuant LAS4000 (GE Healthcare).

### RNA analysis and qPCR

RNA was extracted using RNeasy Mini Kit (Qiagen) and reverse transcription was performed using the High Capacity cDNA Reverse Transcription kit (Applied Biosystems) according to the manufacturer’s guidelines. qPCR was performed using the SensiFAST SYBR No-ROX kit (Bioline). qPCR primers were designed using Primer-BLAST^64^ and were synthesised by Invitrogen. Samples were run using the 7900HT Fast Real-Time PCR System (ThermoFisher) in a 384 well format. Primers are listed in Supplementary Methods Table 2.

### Flow cytometry

Cells were prepared for FACS using standardised FACS methods and measured using a BD FACSCalibur flow cytometer (BD Biosciences). Primary antibodies are listed in Supplementary Methods Table 3. Data was analysed using FlowJo 10.0.7 (FlowJo LLC).

### Confocal microscopy

Confocal microscopy was performed using a Zeiss LSM 780 Confocal Microscope (Zeiss) using standard protocols. DAPI was used to stain nuclei. A 60 × oil lens was used with laser strength adjusted based on results from negative/positive controls. Every well imaged had a minimum of 6 images taken. Images were analysed using FIJI^65^.

### Transient transfection

HEK293T were transiently transfected using Lipofectamine LTX (Invitrogen) according to manufacturer’s guidelines (Thermo Scientific) over 48 h. Transfection efficiency was measured by analysing ADGRL4/ELTD1 expression by qPCR, Western blotting and FACS.

### Signalling assays

In brief, constructs were transiently transfected into HEK293T cells (co-transfection was performed when using the luciferase reported assays) and assayed at 48 h followed by FACS to determine ADGRL4/ELTD1 surface expression. For the *Stachel* peptide experiments in HEK293T cells, cells were transiently transfected with co FL ADGRL4/ELTD1 for 48 h before being exposed to the ADGRL4/ELTD1 *Stachel* peptides for 30 min and commencing the signalling readout assays.

### HTRF FRET assays

Homogeneous Time-Resolved Fluorescence Resonance Energy Transfer (HTRF FRET) assays were performed for Gαq, Gαs and Gαi signalling. For Gαq signalling, Cisbio’s IP-1 assay (Cisbio) was performed. For Gαs and Gαi signalling, Cisbio’s cAMP assay (Cisbio) was performed. At 48 h post transfection, the HTRF FRET assay was run. For the Cisbio IP-1 assay, this involved adding the IP-1 stimulation buffer for 30 min followed by 60 min exposure to the IP1-d2 working solution and anti IP1-cryptate working solution for 60 min. For the Cisbio cAMP assay (for both Gαs and Gαi), this involved adding the cAMP stimulation buffer for 30 min followed by 60 min exposure to the cAMP-d2 working solution and cAMP-Cryptate working solution. For the cAMP assay (encompassing both Gαs and Gαi assays), the phosphodiesterase inhibitor 3-isobutyl-1-methylxanthine (IMBX) was added to the medium at a final concentration of 0.5 mM in order to prevent cAMP degradation. Forskolin was also added at a final medium concentration of 10 μM to the Gαi version of the Cisbio cAMP assay. All HTRF FRET assays were read with a HTRF compatible BMG CLARIOstar microplate reader (BMG Labtech).

### Luciferase reporter assays

In order to classify and compartmentalise experiments, the following luciferase-reporter assays (Promega) were performed as per the manufacturer’s protocols in order to achieve a broad overlapping overview on the following signalling pathways: (1) NFAT-luciferase and NFκB-luciferase for Gαq; (2) SRF-RE-luciferase for Gα12/13; (3) CRE-luciferase and CREB-luciferase for Gαs and Gαi; and (5) SRE-luciferase for Gα12/13, Gαi; Gβγ. Assays were read using a BMG FLUOstar (BMG Labtech). For the NFAT-luciferase assay, a HEK293 cell line stably expressing NFAT-luciferase (Promega) was used thus only requiring transfection with the GPCR plasmid. For the remainder of the luciferase reporters, dual transfection was performed (GPCR plasmid plus reporter plasmid). Constitutively active GPCRs as well as inducer compounds included in the protocols for each luciferase plasmid were used as positive controls. In brief, ELTD1 or control GPCR constructs plus reporter plasmids were transiently transfected into the HEK293T cells or COS-7 cells. For SRE-luciferase and SRF-RE-luciferase, DMEM with 5% FBS was removed and replaced with DMEM with 0.5% FBS (serum starved) 4 h following transfection. On the day of induction, SRE-luciferase transfected cells were stimulated with either an induction solution (40% FBS and 20 ng/ml phorbol myristate acetate (PMA) in DMEM) or a DMEM control solution. For SRF-RE-luciferase transfected cells, an induction solution (40% FBS in DMEM) or a control solution of DMEM was added. For CRE-luciferase transfected cells, an induction solution containing 1 mM forskolin or a control solution of DMEM solution was added. For the above assays, results were read at 5 h. For the NFAT-luciferase assay, an induction solution (1 μM ionomycin and 10 ng/ml PMA) or a control solution (DMSO 1:1000 in DMEM with 5% FBS) was added with results being read 17 h later.

### AlphaScreen assay

PerkinElmer’s AlphaScreen cAMP assay (PerkinElmer) was performed as per the manufacturer’s protocols. In brief, cells were transfected as above. At 48 h, cells were incubated in medium containing IBMX and then lysed in LI buffer (PerkinElmer). Plates were then frozen for 24 h at − 20 °C. The Alpha Screen cAMP assay kit was used to measure cAMP concentration as per the manufacturer’s guidelines. Accumulation was measured in opaque-walled 96 well microplates (PerkinElmer) and read using the PerkinElmer EnVision plate reader (PerkinElmer).

### *Stachel* peptide dilution and application

ADGLR4/ELTD1’s *Stachel* peptides were first diluted with DMSO and then with 50 mM tris(hydroxymethyl)aminomethane (TRIS) (pH8) to yield a final peptide concentration of 1 mM peptide in 1% DMSO (DMSO alone used as control). Peptides were applied to HUVECS and transiently transfected HEK293T cells for 30 min before assessing canonical GPCR signalling activity as described above.

### Cloning and lentiviral transduction

Cloning was performed using the Mammalian Expression System with Gateway Technology (Invitrogen) following the manufacturer’s protocol. In brief, we cloned tagged and untagged codon optimised *FL ADGRL4/ELTD1* into the pLenti6/V5-DEST lentiviral expression vector and assembled a virus-rich supernatant from transfected HEK293T cells, using the ViraPower Lentiviral Expression System (Invitrogen), that was used to transduce 3 differing batches of pooled low-passage HUVECs. After 48 h, media was replaced with EBM-2 supplemented with EGM-2 as well as blasticidin (4 μg/mL) (Gibco) selection. HUVECS were only used within 5 passages.

### Angiogenesis assays

#### Hanging drop endothelial sprouting spheroid assay in matrigel

ADGRL4/ELTD1 overexpressing HUVEC spheroids (~ 500 cells each, 100 per 15 cm dish) were generated using the hanging-drop method (Kelm et al., 2003) and then embedded in matrigel (BD Biosciences). Spheroids were photographed (20 images of each dish) using an AMG Evos XL Core digital microscope (Fisher Scientific) after 24 h and 48 h and a self-made stylus was used to accurately trace the outline of each spheroid which were then analysed using a script in FIJI^65^.

#### Fibrin gel bead assay

This assay was performed as originally described^66^ using ADGRL4/ELTD1 overexpressing HUVECS coated onto cytodex beads which were then embedded in a fibrin gel overlaid with fibroblasts. Medium was changed every 2 days with EGM2 (Lonza) and images were taken every second day. See supplementary methods for further details.

#### Tube formation assay on matrigel

Control and ELTD1 overexpressing HUVECS were plated at 5 × 10^4^ cells/well on top of 200 µl of matrigel in a 24-well plate (BD Biosciences) The plate was then placed within an Incucyte Imaging Incubator (5% carbon dioxide at 37 °C) (Essen BioScience) and 10 photos were taken per well every hour for 24 h. Tube formation was analysed by counting the number of complete polygons (comprising endothelial tubes) in each image field. Results were analysed using FIJI.

#### Endothelial proliferation assay

In this assay, control and ELTD1 overexpressing HUVECS were plated at 1 × 10^5^ cells per well (low confluence) into multiple 12-well plates. Every day, one plate was selected for photographing, harvesting and cell counting. This was repeated until both control and ELTD1 groups achieved confluence. Photographs were performed using an Incucyte Imaging Incubator (5% CO_2_ at 37 °C). Sixteen photographs were taken of each well, then cells were harvested and counted as described in the cell lines and maintenance methods section. Results were analysed using FIJI.

#### Endothelial migration assay

Control and ELTD1 overexpressing HUVECS were seeded at 5 × 10^5^ cells per well into a 24-well ImageLock plate (Essen BioScience) and grown to confluence. Once confluent, a scratch defect was introduced into each well using a 4 pin Essen Wound Maker (Essen BioScience) and scratches were imaged every 30 min, using an Incucyte Imaging Incubator (5% CO_2_ at 37 °C), until the defect had been repaired. The cell-free scratched images of each group at individual time points were then manually traced using the self-made stylus described above with the area quantified using FIJI.

#### MilliCoat extracellular matrix (ECM) adhesion assay

This assay was performed using the MilliCoat ECM Screening Kit (Merck: ECM105) according to the manufacturer’s instructions, seeding 1 × 10^4^ HUVECS into wells coated individually with the various ECM adhesion components (96-well format). Absorbance was read at 560 nm using a FLUOstar optima microplate reader (BMG Labtech). microplate reader. Results were analysed using FIJI.

#### Matrigel adhesion assay

Following matrigel solidification, control and ELTD1 overexpressing HUVECS were seeded at 1 × 10^4^ cells into each well of a 96-well plate. After 1 h cells were washed 3 × with warmed PBS and the centre of each well was photographed using an AMG Evos XL Core digital microscope (Fisher Scientific). Images were analysed using FIJI.

## Microarray analysis

Gene expression profiling analysis of ADGRL4/ELTD1 overexpressing HUVECs was performed by the Wellcome Trust Centre for Human Genetics (Oxford). Microarrays were performed by reverse transcribing extracted mRNA into cDNA, labelling the cDNA, and then hybridising the labelled target to the microarray chip. Raw data outputted from the Illumina microarray scanner (Illumina iScan) using a HumanHT-12 v4 Expression BeadChip Kit (Illumina) was analysed using using R (version 3.3.2) and Limma^67^. Differentially expressed genes were deemed statistically significant if they fulfilled standard microarray criteria: (1) an adjusted *p* value < 0.05; and (2) a fold change of > 2 < − 2. Visualisation was performed using “GGplot2”^68^. All microarray experiments were MIAME compliant^69^.

## Statistical analyses using Prism

Prism 7 (Graphpad Software) was used to analyse results from all completed wet-lab experiments. For comparison of two unpaired groups, the unpaired Student's *t*-test was used. For comparison of two or more groups, a one-way ANOVA was computed. Significance is denoted as: *p ≤ 0.05, **p ≤ 0.01, ***p ≤ 0.001, ****p ≤ 0.0001.

## Supplementary Information

Supplementary Figures and Tables
