# Supplementary material for: Elevated expression of the adhesion GPCR ADGRL4/ELTD1 promotes endothelial sprouting angiogenesis without activating canonical GPCR signalling
Source: Sci Rep. 2021 Apr 23;11:8870. doi: 10.1038/s41598-021-85408-x (PMC8065136; doi:10.1038/s41598-021-85408-x)
Supplement: Supplementary file 1 — Supplementary Figures and Tables [file 41598_2021_85408_MOESM1_ESM.docx]

**Title:** Elevated expression of the adhesion GPCR ADGRL4/ELTD1 promotes endothelial sprouting angiogenesis without activating canonical GPCR signalling

**Author list:** David M Favara^1,2*$^, Ines Liebscher^3^, Ali Jazayeri^4#^, Madhulika Nambiar^4§^, Helen Sheldon^2^, Alison H Banham^5^, Adrian L Harris^2*^

^1^ Balliol College, University of Oxford, Oxford, OX1 3BJ, United Kingdom,

^2^ Department of Oncology and Weatherall Institute of Molecular Medicine, University of Oxford, Oxford, OX3 7DQ United Kingdom. $Currently: Cambridge University Hospitals NHS Foundation Trust and Department of Oncology, Cambridge University, Cambridge, CB2 0XZ, United Kingdom.

^3^ Rudolf Schönheimer Institute of Biochemistry, Department of Molecular Biochemistry, University of Leipzig, Leipzig, 04103, Germany

^4^ Heptares Therapeutics Ltd, Welwyn Garden City, AL7 3AX, United Kingdom. #Currently: OMass Therapeutics, Oxford, OX4 4GE, United Kingdom. §Currently: Sosei Heptares, Cambridge, CB21 6DG, United Kingdom.

^5^ Nuffield Division of Clinical Laboratory Science, Radcliffe Department of Medicine, University of Oxford, Oxford, OX3 9DU, United Kingdom

* corresponding authors: df411@cam.ac.uk / dmf@dmf.co.za; adrian.harris@oncology.ox.ac.uk

**Supplementary Images:**

**Figure S1.** ADGRL4/ELTD1 codon optimisation and construct expression. **(A)** wild type and co ADGRL4/ELTD1’s GC content compared to members of the adhesion and secretin GPCR families. **(B)** Representative FACS histograms of HA tagged (surface expression level) and GFP tagged (whole cell fluorescence level) ADGRL4/ELTD1 signalling constructs in transfected HEK293T cells. **(C)** Representative FACS plots of GFP tagged ADGRL4/ELTD1 comparing total GFP expression between wt and co constructs in transfected HEK293T cells. **(D)** Representative confocal microscopy of GFP C-terminal tagged co FL and CTF ADGRL4/ELTD1 constructs expressed by transfected HEK293T cells. **(E)** ELISA surface expression comparison between ADGRL4/ELTD1’s constructs and FL and CTF forms of ADGRG6, and P2Y_12_. **(F)** Gαs AlphaScreen cAMP assay in transiently transfected HEK293T cells. forskolin and the FL and CTF forms of ADGRG6/GPR126 were used as positive controls (*p* < 0.0001). (Abbreviations: Pos CT=positive control; co=codon optimised; CTF=C-terminal fragment; FL=full length; FSK=forskolin; Neg CT=negative control; GFP=green fluorescent protein; HA=hemagglutinin; wt=wild type)

**Figure S2.** ADGRL4/ELTD1 *Stachel* peptide signalling experiments in HEK293T and endothelial cells. **(A)** Gαs and Gαi HTRF FRET assays in co FL ADGRL4/ELTD1 transfected HEK293T cells. **(B)** Gαs and Gαi HTRF FRET assays in HUVECs. (Abbreviations: HTRF=homogenous time resolved fluorescence; FRET=fluorescence resonance energy transfer; co=codon optimised; FL= full length; HA=hemagglutinin; HUVEC=human umbilical vein endothelial cell).


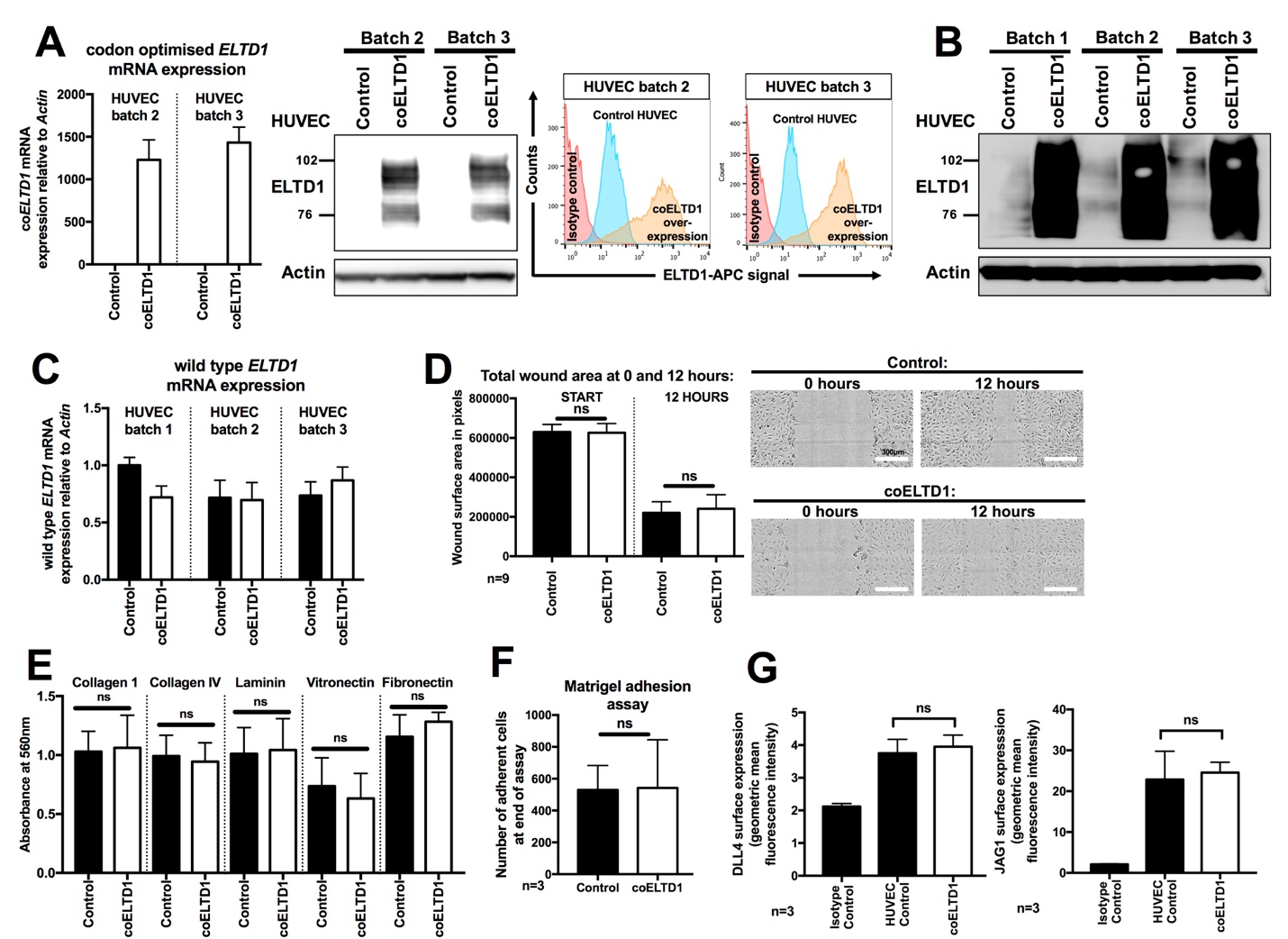


**Figure S3.** ADGRL4/ELTD1 overexpression in HUVECs**. (A)** Expression of codon optimised FL ADGRL4/ELTD1 in HUVEC batches 2 and 3 (mRNA, total protein and surface protein level). **(B)** Representative overexposed Western blot for all 3 HUVEC batches showed ADGRL4/ELTD1 in the control HUVEC groups. **(C)** qPCR showed similar levels of wild type *ADGRL4/ELTD1* mRNA in both control and FL ADGRL4/ELTD1 transduced HUVECs. Overexpression of ADGRL4/ELTD1 did not affect **(D**) endothelial migration**; (E)** adherence to angiogenesis-associated ECM components**; (F)** HUVEC adhesion to matrigel**;** or **(G)** cell surface DLL4 protein expression.

**
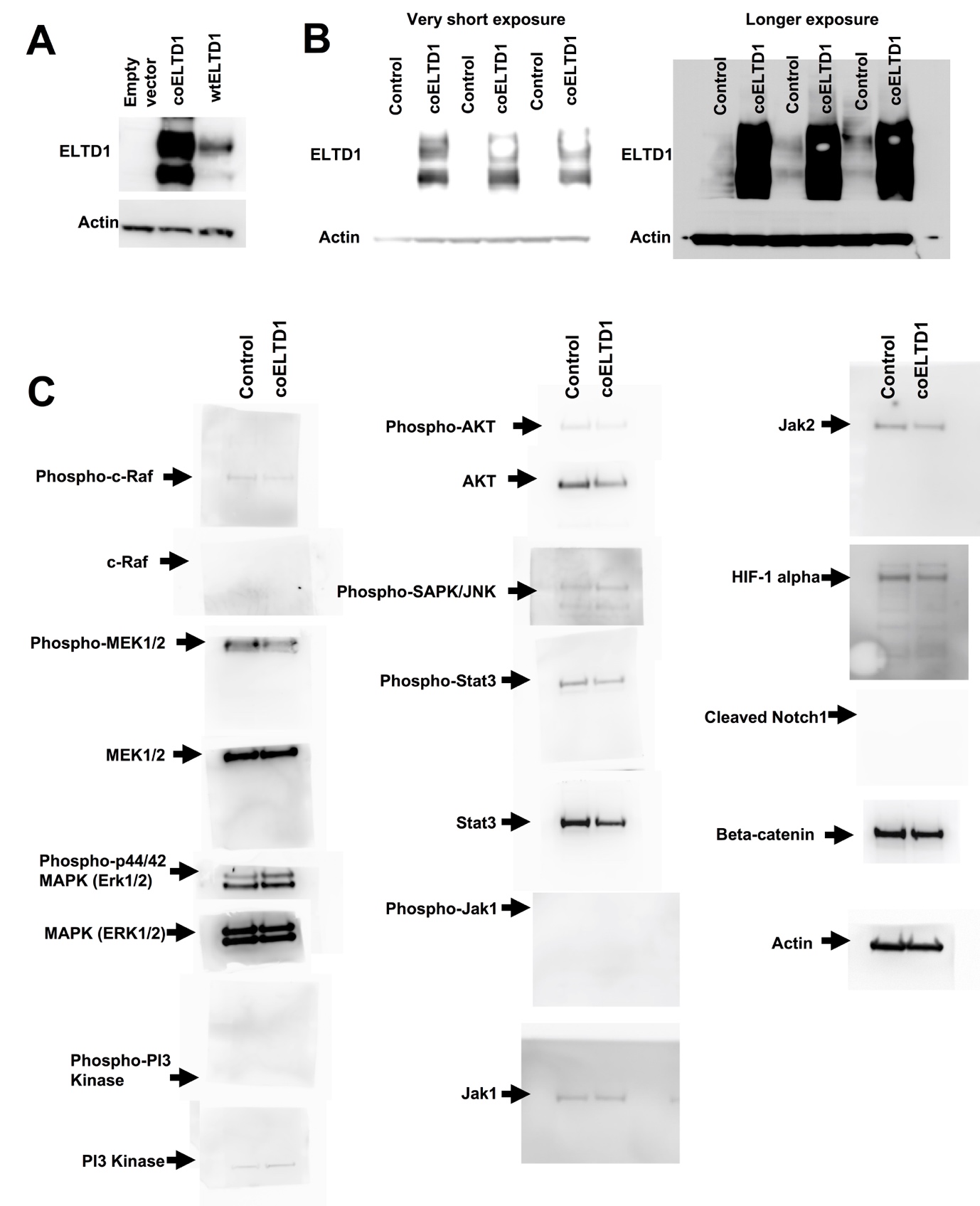
Supplementary figure of uncropped western blots**. **(A)** Western blot featured in Figure 1A. **(B)** Western blot featured in Figure 3B, Supplementary S3A, Supplementary S3B. **(C)** Western blots featured in Figure 4B

**Supplementary Methods:**

#### **Fibrin gel bead assay (Additional methods):**

Cytodex beads (Amersham) were prepared as follows: 0.5 g of dry beads were hydrated using 50 ml PBS (pH 7.4) for 3 hours at room temperature on a rocker. Following this, supernatant was discharged and beads were washed 3 times in 50 ml of fresh PBS yielding 10 mg/ml of beads (approximately 30000 beads/ml). Bead suspension was then placed in a glass bottle, autoclaved and kept at 4 °C. The day before the assay, the beads were coated with HUVECS. This was performed by adding 2500 beads to 1x10^6 HUVECS in 1.5 ml of warm EGM2 within a FACS tube (Corning). These were incubated in an aseptic humidified incubator (5% carbon dioxide at 37°C) for 4 hours. During this period, each FACS tube was manually tapped/shaken every 20 minutes to prevent clumping. Following 4 hours, the coated beads were transferred to an upright T25 size flask in 5 ml of EGM2 with flasks left overnight in an upright position within an aseptic humidified incubator (5% carbon dioxide at 37°C). The following day, HUVEC coated beads were transferred to a 15mL tube (Corning). Once beads had settled, media was removed and beads washed gently three times with 3 ml of EGM2. Thereafter, beads were counted by suspending beads on a slide (in triplicate) and counted using a haemocytometer. Following this, 1500 beads were added to a FACS tube (Corning) and all media was removed once beads had settled to bottom of tube. Following this, the beads were suspended in 3 ml of 2 mg/ml fibrinogen solution. 0.625 units/ml thrombin (Sigma-Aldrich) was then added to each well of the 24 well plate. Thereafter, 0.5 ml of the fibrinogen-HUVEC bead solution was added to each well. This was followed by clotting and an incubation of 15 minutes in an aseptic humidified incubator (5% carbon dioxide at 37°C). Once clotting occurred, 1ml of EGM2 medium and 20 000 low passage fibroblasts (Lonza) were added to each well. Medium was changed every 2 days and photos were taken using an AMG Evos XL Core digital microscope (Fisher Scientific) over a 3-week period.

|  | Amino acid sequences: |
| --- | --- |
| 1 | THFAILMSSGPSIGIKDYNILTRI |
| 2 | THFAILMSSGPSIGIKDYNILTR |
| 3 | THFAILMSSGPSIGIKDYNILT |
| 4 | THFAILMSSGPSIGIKDYNILT |
| 5 | THFAILMSSGPSIGIKDYNIL |
| 6 | THFAILMSSGPSIGIKDYNI |
| 7 | THFAILMSSGPSIGIKDYN |
| 8 | THFAILMSSGPSIGIKDY |
| 9 | THFAILMSSGPSIGIKD |
| 10 | THFAILMSSGPSIGIK |
| 11 | THFAILMSSGPSIGI |
| 12 | THFAILMSSGPSIG |
| 13 | THFAILMSSGPSI |
| 14 | THFAILMSSGPS |
| 15 | THFAILMSSGP |
| 16 | THFAILMSSG |
| 17 | THFAILMSS |
| 18 | THFAILMS |
| 19 | THFAILM |
| 20 | THFAIL |
| 21 | THFAI |
| 22 | THFA |

**Supplementary Methods table 1** ADGRL4/ELTD1 Stinger/Stachel peptide sequences

| **Gene name** | **Forward primer (5’-3’)** | **Reverse primer (5’-3’)** |
| --- | --- | --- |
| ACTB | ATGCTCTCCCTCACGCCATC | CACGCACGATTTCCCTCTCA |
| ANTXR1 | GACAAGGGTACAGGACAGCC | CTCCCCAACGAACCTCCATT |
| ANTXR1 | TTTGAAAGGGCCAGTGAGCA | TCCCCGTTTTCAGGAGTGTG |
| ANTXR1 | CCACACACTGTTCTGACGGT | GCACGGGCTGTGTTAGGTTA |
| CCL15 | GCCCAGTTCACAAATGATGCAG | AACTCACAGGAGGTGTTGGAG |
| CHRNA1 | GGACCTACGACGGCTCTGT | GCTCTCCATGAAGTTGCTCAG |
| CYP26B1 | GCCAACATGCTCTTTGAGGG | GAGAAGACCTTGCGCTTGTTG |
| CYP26B1 | TGCTGCAGGTCTTCTCCAAG | GCAAAGATCAGCTCCAGGGT |
| DLL4 | CCCTGGCAATGTACTTGTGAT | TGGTGGGTGCAGTAGTTGAG |
| ELMOD1 | ACTGTTCCAAAAGAAGACGTCAG | AGTGATATCCCTGCATTTCGGA |
| ELMOD1 | GCACTTCCTGAGAATGTTGATCC | TCCATGGGGTCCTCTTCGAT |
| ELMOD1 | CCAGGGAAGCCTCTGCAC | AGTGTCAACTGGACTCTGGC |
| ADGRL4/ELTD1 (codon optimised) | GGCCTGCTGCACTACTTCTT | AGGTGGATGCCCTCGATAC |
| ADGRL4/ELTD1 (wild type) | GCTCAAACCCACCCACATTAT | CACAGCCCTCTGAAGACCAG |
| GAPDH | AGCCACATCGCTCAGACAC | GCCCAATACGACCAAATCC |
| HBB | ACTTCAGGCTCCTGGGCAAC | GGCCCTTCATAATATCCCCCAG |
| JAG1 | GAATGGCAACAAAACTTGCAT | AGCCTTGTCGGCAAATAGC |
| KIT | AATCCTCTCGTCAAAACTGAAGG | CCATCTCGCTTATCCAACAATGA |
| SLC39A6 | CCAACCCAATGAAACCACCG | CATTGCGCCTTCGTCTCCG |
| SLC39A6 | CCGCGGAGACTGTTTCAATG | GCTCTTGTGAGTCTGCTCGT |

**Supplementary Methods table 2** qPCR primer sequences.

Repeated genes indicate qPCR primers designed to probe all splice variants of a given gene.

| **Target** | **Type** | **Dilution** | **Company** |  |  |
| --- | --- | --- | --- | --- | --- |
| **Primary antibodies** | | | | |  |
| ADGRL4/ELTD1 | Mouse IgG2 monoclonal | 10 μg/mL | Professor Alison Banham, Oxford University (184.11) |  |  |
| HA tag | Mouse IgG1 monoclonal | 1 in 50 | Cell Signaling Technology (2367) |  |  |
| DLL4 | Rat IgG2 monoclonal | 10 μg/mL | R&D Systems (MAB1506) |  |  |
| JAG1 | Mouse IgG2 monoclonal | 1:200 | Professor Alison Banham, Oxford University (JAG1) |  |  |
| KIT | Mouse IgG1 monoclonal | 1:200 | Invitrogen (17-1178-41) |  |  |
| IgG1 isotype control | Mouse IgG1 monoclonal | 10 μg/mL | Invitrogen (17-4714-41) |  |  |
| IgG2 isotype control | Mouse IgG2 monoclonal | 10 μg/mL | Invitrogen (17-4724-41) |  |  |
| **Secondary antibodies** | | | | |  |
| anti-mouse APC | Goat IgG | 1 in 100 | Invitrogen (A-865) |  |  |
| anti-rat APC | Goat IgG | 1 in 100 | Invitrogen (A10540) |  |  |

**Supplementary Methods table 3** Antibodies used for cell surface FACS.
